# Editorial: The impact of age-related changes in brain network organization and sleep on memory

**DOI:** 10.3389/fnagi.2022.1049278

**Published:** 2022-10-04

**Authors:** Alison Mary, Christine Bastin, Jean-Marc Lina, Géraldine Rauchs

**Affiliations:** ^1^UR2NF—Neuropsychology and Functional Neuroimaging Research Unit at CRCN - Center for Research in Cognition and Neurosciences and UNI—ULB Neuroscience Institute, Université Libre de Bruxelles (ULB), Brussels, Belgium; ^2^GIGA-Cyclotron Research Centre In Vivo Imaging & Psychology and Neuroscience of Cognition, University of Liège, Liège, Belgium; ^3^Center for Advanced Research in Sleep Medicine, Hôpital du Sacré-Coeur de Montréal, Montreal, QC, Canada; ^4^Department of Electrical Engineering, École de Technologie Supérieure, Montreal, QC, Canada; ^5^Centre de Recherches Mathématiques, Université de Montréal, Montreal, QC, Canada; ^6^Normandie Univ, University of Caen, Institut national de la santé et de la recherche médicale, U1237, Physiopathology and Imaging of Neurological Disorders (PhIND), GIP Cyceron, Institut Blood and Brain @ Caen-Normandie, Caen, France

**Keywords:** aging, sleep, memory, brain network, mental health, hormones

As the worldwide population is rapidly aging, there is a higher prevalence of age-related cognitive disorders that alter quality of life. Memory decline is the most frequent complaint in the elderly (Grady, 2012), and it is accompanied by changes in brain network organization (Persson et al., 2014; Bernard et al., 2015; Fjell et al., 2015a,b) and in sleep (for reviews, see Harand et al., 2012; Pace-Schott and Spencer, 2014). Aging is characterized by lower within- and higher between-network connectivity that suggest less segregated brain networks (for a review, see Damoiseaux, 2017). Both the macro- and micro- structure of sleep changes drastically with aging (Ohayon et al., 2004; Scullin and Bliwise, 2015; Mander et al., 2017; André et al., 2021) and sleep disruption may increase the risk of developing neurodegenerative diseases (Ju et al., 2014; Nedergaard and Goldman, 2020; Winer et al., 2020). Understanding the neurophysiology of sleep and the spatiotemporal organization of brain networks in aging is critical to predict who is at greater risk of cognitive decline and to develop effective interventions to maximize cognitive function and wellbeing across lifespan. This Research Topic aimed at providing an updated view on how age-related changes in brain and sleep mechanisms may affect cognition in healthy aging and neurodegenerative processes.

Several studies evidenced that self-reported sleep disruption (i.e., shorter or longer sleep duration, greater sleep fragmentation) are associated with cognitive decline (Lo et al., 2016; Winer et al., 2021) including memory impairment (Mary et al., 2013; Lo et al., 2016), and with an increased risk of incident dementia at older age (Spira et al., 2013; Branger et al., 2016; Sabia et al., 2021; Winer et al., 2021). In line with these studies, Bubu et al. reported that self-reported sleep disturbance as well as vascular risk factors increase the likelihood of prospective cognitive decline in older adults, even after adjusting for some biomarkers of Alzheimer's disease (i.e., amyloid-β and Tau levels in cerebrospinal fluid, hippocampal volume). However, this relationship between sleep disruption and decreased cognition is not limited to older adults. Federico et al. showed that poorer sleep quality in adults (range: 20–68 years old) is associated with functional connectivity (FC) changes in limbic and fronto-temporo-parietal brain regions, increased symptoms of depression and anxiety, and decreased visuospatial working memory performance. Tibon and Tsvetanov demonstrated, in a large cohort of 564 healthy adults (range: 18–88 years old), that increased subjective sleep dysfunction and decreased fluid intelligence is associated with a shift in brain network dynamics, characterized by an increased occurrence of brain states involving “higher-order” fronto-temporo-parietal networks and a reduced occurrence of “lower-order” visual network. These results are congruent with previous studies showing that sleep disruption in older adults is associated with an increased risk of depression and anxiety (Potvin et al., 2014) and with changes in the fronto-temporo-parietal network (André et al., 2021). Psycho-affective symptoms (Harrington et al., 2015; Kuring et al., 2020; Moulinet et al., 2022) and sleep disturbances (Mander, 2020; Winer et al., 2020) are both risk factors for the progression to neurodegenerative diseases, such as Alzheimer's disease. At the clinical level, these findings highlight the importance to screen older adults for both sleep disruption and neuropsychiatric symptoms to improve the prevention and the early detection of cognitivedecline.

Self-reported sleep measures can be an easy and cost-effective predictor of the potential evolution toward pathological aging in epidemiological studies. However, subjective sleep measures have been modestly correlated with objective measures such as actigraphy or polysomnography (Landry et al., 2015; Matthews et al., 2018) and can be influenced by the presence of cognitive decline or negative affects such as depressive symptoms (Matthews et al., 2018). Objective measures can therefore more directly shed light on how sleep benefits memory processes. At the system level, memory consolidation involves an active reinstatement of memory-related brain networks during the subsequent sleep period (Peigneux et al., 2004; Bergmann et al., 2012; Fogel et al., 2017), but also during post-training quiet resting-state (Tambini et al., 2010; Vahdat et al., 2011; Jacobs et al., 2015; Mary et al., 2017). Fang et al. showed that a daytime nap strengthens resting-state FC within the striato-cortico-hippocampal network after motor learning in young adults, whereas this FC is decreased in older adults. Faßbender et al. evidenced that brain reorganization between large-scale networks (i.e., salience, central executive, and default mode networks) following episodic memory encoding becomes less predictive of memory performance with age, and their dynamics is sensitive to retroactive interference in older participants. These results corroborate the idea that aging is associated with a decreased segregation of functional networks, which may impair learning and memory as shown in previous studies (King et al., 2017; Mary et al., 2017; Cassady et al., 2021). At the synaptic level, local brain activity during learning can trigger local learning-dependent increase in NREM sleep oscillatory activity (Huber et al., 2004; Krueger et al., 2008; Mascetti et al., 2013; Tamaki et al., 2013). In aging, local sleep following motor sequence learning is reduced in delta, theta, and sigma frequency bands in memory-related brain regions (Fitzroy et al.). Local sleep (i.e., frequency slowing) can also occur during quiet wakefulness following a memory task and it is associated with memory improvement in both young (Brokaw et al., 2016) and older (Sattari et al., 2019) adults.

It is important to note that the prevalence of dementia is higher in women than in men and this is not only due to increased longevity (Mazure and Swendsen, 2016). In an extensive literature review, Harrington et al. highlighted that fluctuations of estradiol and progesterone levels across menstrual cycle, pregnancy and menopausal transition may alter sleep and memory. Menopausal transition is associated with greater risk of memory decline and sleep disturbance and could therefore be a critical period for intervention (Baker et al., 2019; Brown and Gervais, 2020). Further studies are needed to understand how hormonal changes across women lifespan affect sleep and cognitive trajectories in aging.

To conclude, brain integrity and sleep are crucial for preserving and optimizing cognitive function and quality of life with advancing age. The strong association between sleep disruption and cognitive decline highlights the importance of maintaining adequate sleep throughout life, not solely at advanced age. Mental health factors such as anxio-depressive symptoms can also alter the impact of age on sleep and memory and should be better scrutinized in aging studies. Moreover, sex differences and hormonal fluctuations across women lifespan should be considered when investigating brain functions, sleep and cognition. Hormones, sleep, and mental health throughout life are modifiable factors that can be targeted for intervention to reduce the risk of neurodegenerative diseases later in life. Identifying the risk and protective factors of neurocognitive aging have important implications to develop effective interventions and preventive measures to promote successful aging. However, there is a large inter-individual variability in the neurocognitive trajectories in aging. It remains a fundamental challenge to distinguish brain and sleep changes that are specific to normal or pathological aging. In this context, methodological advances in neuroimaging are still needed to provide a more quantitative time-resolved description of the functional networks dynamics. Such advancements should emphasize the neural plasticity at various scales of the processes integrated in dynamical networks, in order to better understand the neurobiology of cognitive aging.

## Author contributions

AM wrote the first draft of the editorial. All authors contributed to manuscript revision, reading, and approved the submitted version.

## Funding

AM and CB are supported by the Fonds de la Recherche Scientifique-FNRS (AM: FRS-FNRS grant reference 1226419F). J-ML is supported by a NSERC-Discovery grant. GR is supported by the Association France Alzheimer (AAPSM2017, grant 1714).

## Conflict of interest

The authors declare that the research was conducted in the absence of any commercial or financial relationships that could be construed as a potential conflict of interest.

## Publisher's note

All claims expressed in this article are solely those of the authors and do not necessarily represent those of their affiliated organizations, or those of the publisher, the editors and the reviewers. Any product that may be evaluated in this article, or claim that may be made by its manufacturer, is not guaranteed or endorsed by the publisher.
